# Exergame Grading Scheme: Concept Development and Preliminary Psychometric Evaluations in Cancer Survivors

**DOI:** 10.1155/2017/6843016

**Published:** 2017-10-02

**Authors:** Hsiao-Lan Wang, Chiung-Ju Liu, Marcus Kilpatrick, Heather Jim, Susan McMillan, Nisha Vijayakumar, Sally McDonald, Tapan Padhya, Jeffery Russell, Karen Vondruska, Constance Visovsky

**Affiliations:** ^1^College of Nursing, University of South Florida, Tampa, FL, USA; ^2^School of Health and Rehabilitation Sciences, Indiana University, Indianapolis, IN, USA; ^3^College of Education, University of South Florida, Tampa, FL, USA; ^4^Moffitt Cancer Center, Tampa, FL, USA; ^5^College of Public Health, University of South Florida, Tampa, FL, USA; ^6^College of Medicine, University of South Florida, Tampa, FL, USA

## Abstract

The challenge of using exergames to promote physical activity among cancer survivors lies in the selection of the exergames that match their fitness level. There is a need for a standardized grading scheme by which to judge an exergame's capacity to address specific physical fitness attributes with different levels of physical engagement. The study aimed to develop an Exergame Grading Scheme and preliminarily evaluate its psychometric properties. Fourteen (14) items were created from the human movement and exergame literature. The content validity index (CVI) was rated by content experts with two consecutive rounds (*N* = 5 and *N* = 3 independently). The interrater reliability (IRR) was determined by two raters who used the Exergame Grading Scheme to determine the grading score of the five exergames performed by two cancer survivors (*N* = 10). Each item had a score of 1 for item-level CVI and 1 for *k*. For IRR, 9 items had rho values of 1, 1 item had 0.93, and 4 items had between 0.80 and 0.89. This valid and reliable Exergame Grading Scheme makes it possible to develop a personalized physical activity program using any type of exergame or fitness mobile application in rehabilitation practice to meet the needs of cancer survivors.

## 1. Introduction

One of every 25 Americans is a cancer survivor [1]. A cancer survivor, based on the definition of the American Cancer Society (ACS), is an individual who has been diagnosed with cancer from the time of diagnosis to the end of life [2]. Currently, about 4.7 million cancer survivors have lived 10 years or more after diagnosis. As the increased numbers of cancer survivors are living longer, a physically active lifestyle after their cancer diagnosis has been recommended by the ACS and the American College of Sports Medicine (ACSM) [3, 4]. This recommendation was based on systematic reviews or meta-analyses showing positive outcomes of physical activity among cancer survivors in terms of reduced cancer recurrence and improved overall cancer survival rates, enhanced physical fitness, reduced cancer-related symptoms, and better quality of life [5–10]. However, it has been shown that most cancer survivors maintain a sedentary lifestyle or actually reduce their physical activity after their cancer treatment [11].

Novel applications of technology that promote physical activity have been studied in relation to the many different disease conditions. The most frequently applied technology, the exergame (e.g., Nintendo Wii Sport and Wii Fit), has been found to increase physical activity among adults with chronic diseases such as Parkinson's disease [12], stroke [13], multiple sclerosis [14], cerebral palsy [15], chronic obstructive pulmonary disease [16], and lupus [17]. A recent study showed that there was a high acceptance rate for Wii games among adult cancer patients during hospitalization [18]. Among postthoracotomy lung cancer patients, the adherence rate was above 85% for prescribed walking and balance activities with Wii exergames at home [19, 20].

There are several benefits to be gained from the use of exergames in terms of promoting physical activity [21]. First, exergames are designed to be enjoyable and motivating, so users may increase the amount of time and energy they expend in physical activity while engaging in an exergame. Second, visual feedback on postural control and accuracy is provided during performance of most exergame physical activities, and overall competency is indicated upon completion of an exergame. Therefore, exergames may be used to improve specific physical fitness attributes that are related to the ability to perform human movement (i.e., physical activity) such as cardiorespiratory fitness [14, 19], muscular strength [16], flexibility [13], and balance [14, 22, 23]. Third, exergames are accessible and relatively affordable, so they can be used in a wide range of environments, including homes [14, 19], clinical sites [18], and residential care settings [22].

Cancer survivors prefer to engage in physical activities personalized to their fitness level since fitness capacity varies by cancer type and cancer treatment and is also influenced by the physiological aging process [24]. The challenge of using exergames to promote physical activity among survivors lies in the appropriate selection of the exergame to match the fitness level [12, 25]. Thus, there is a need for a standardized grading scheme by which to judge the exergame's capacity to address specific physical fitness attributes with different levels of physical engagement. A standardized grading scheme allows for between-study comparisons of research using the same technology. Rehabilitation professionals can utilize the grading from this scheme to determine the appropriate exergames for a cancer survivor and therefore to design a personalized physical activity prescription.

The purpose of this study was to develop an Exergame Grading Scheme and its content validity index (CVI). We also used Wii Fit U exergames (Nintendo, Redmond, WA, USA) in evaluating the interrater reliability (IRR) of the Exergame Grading Scheme. There were two hypotheses guiding the psychometric evaluation of the Exergame Grading Scheme: (a) For the CVI, the kappa statistic for each item in the Exergame Grading Scheme would be at least 0.74, which is considered to be excellent content validity [26] and (b) for the IRR, Spearman's rho for each item in the Exergame Grading Scheme would be at least 0.8 as a value of acceptable reliability [27].

## 2. Methods

The current study was approved by the University of South Florida institutional review board. Two types of human subjects were included in the study. First, content experts that contributed the CVI participated in the study anonymously along with a waiver of informed consent. Second, cancer survivors gave their written informed consent for participation in the IRR test.

### 2.1. Content Validity

The current study used the two-stage process to develop the content validity, as described by Lynn [28]. The first stage was a development stage: identifying the domain of content through a comprehensive literature review, generating items related to the domains, and adding respondents and scoring mechanisms. The second stage was a judgment/quantification stage to evaluate the CVI.

#### 2.1.1. Stage 1: Development of the Exergame Grading Scheme

The first step in the development of the Exergame Grading Scheme was to create a set of domains using the constructs in the concept of human movement [21, 29] as well as the findings from previous exergame studies [12, 30]. After the domains were identified, conceptual and operational definitions of the items were created and then reviewed by an expert panel, which included an exercise physiologist, an occupational therapist, a cancer researcher, and two senior oncology nursing scientists with specialties in instrument design and exercise intervention. Patients were not included in this panel because the Exergame Grading Scheme was designed for rehabilitation personnel who would make physical activity prescriptions for patients.

*Descriptions of Domains.* Seven domains were created. Exergames instruct individuals to perform specific physical activities [31]. Each physical activity requires a certain level of “energy expenditure” (domain 1) and five physical fitness attributes to complete: “cardiorespiratory fitness” (domain 2), “muscular strength” (domain 3), “muscular endurance” (domain 4), “flexibility” (domain 5), and “balance” (domain 6) [21]. Exergames are designed to use game stimuli and game reinforcements to shape game behavior, which is physical activity [31]. Therefore, “cognitive demand” (domain 7) to respond to stimuli and reinforcement is also an important factor in the completion of the physical activity tasks in the exergames.

*Conceptual and Operational Definitions of Items for Each Domain.* From the seven domains, 14 items were developed in the Exergame Grading Scheme. Their conceptual and operational definitions are described as follows.

*Energy Expenditure (Item 1).* Energy expenditure is conceptualized as the total amount of energy required by an individual to perform a physical activity [21]. In the Exergame Grading Scheme, energy expenditure is operationalized by one item that records the Metabolic Equivalent of Task (item 1: MET) required for engaging in a specific exergame. The number of METs is the ratio of the rate of energy expended during a physical activity to the rate of energy expended at rest [29]. One MET is equal to the resting metabolic rate. The calories spent on one exergame vary based on body weight and time (i.e., MET = kcal·kg^−1^·h^−1^). Wii Fit U provides MET numbers for each exergame but does not explain the method used to derive these values [25]. Researchers used open-circuit indirect metabolic chambers to measure the METs of some aerobic and balance exergames in Wii Fit U [32]. Participants in this study were healthy men and women aged between 30 and 45 years. The results of the METs were similar to those posted in Wii Fit U. For example, the exergame, “Rowing Crew,” generated a mean of 3.0 METs from the study, which is the same number of METs posted in Wii Fit U; “Puzzle Squash” was 3.3 METs in the study and 3.5 METs in Wii Fit U.

*Cardiorespiratory Fitness (Items 2 and 3).* Cardiorespiratory fitness is conceptually defined as the ability of the circulatory and respiratory systems to supply oxygen during sustained physical activity and is measured by maximal oxygen consumption (VO_2max_) [29]. Use of VO_2max_ as an operational definition for cardiorespiratory fitness is not feasible and may not be accurate among individuals with chronic disease or deconditioning. An alternative to VO_2max_ that describes the cardiorespiratory response and can be more easily qualified is the heart rate reserve (HRR). This approach considers both age-predicted maximal heart rate (HR_MAX_ = 220 − age) and resting heart rate (HRR = HR_MAX_ − resting  heart  rate). In the Exergame Grading Scheme, cardiorespiratory fitness is operationalized in two items: percentage of HRR (item 2: HRR%) and perceived exertion (item 3: PE-CR). For item 2, the percentage of HRR is calculated by finding the difference between resting heart rate and the peak heart rate during the exergame activity, then dividing by HRR, and multiplying by 100 [29]. For example, a 55-year-old individual has a resting heart rate of 70 bpm and a peak heart rate during an exergame of 120 bpm. The HRR is 95 bpm (220 – 55 y/o – 70 bpm). The percentage of HRR elicited from the exergame is 53% ([120 − 70]/95 × 100%). For item 3, perceived exertion in cardiorespiratory fitness in the Exergame Grading Scheme is operationalized by the Adult OMNI Scale of Perceived Exertion for the Walking/Running Exercise [33]. This scale asks about subjective feelings of peak maximal exertion of the activity and ranges from 0 to 10, with words anchored at “0” (extremely easy), “2” (easy), “4” (somewhat easy), “6” (somewhat hard), “8” (hard), and “10” (extremely hard). Individuals are asked to report their perceived exertion by selecting a number that is consonant with the score depicted visually by a figure walking at the bottom (rating 0) and top (rating 10) of a hill as presented in the scale. Its concurrent validity with oxygen uptake, relative maximal oxygen uptake, minute ventilation, respiratory rate, and heart rate during a graded exercise test on a treadmill has been well studied (*r* = 0.67–0.88, *p* < 0.05) [33].

*Muscular Strength (Items 4, 5, and 6).* Muscular strength is one of two components of muscular fitness [21]. Its conceptual definition is the force that can be exerted by a specific muscle or muscle group to go against resistance [29]. Most of the exergames, especially the Wii Fit U exergames, do not use external weight as the source of resistance. To determine the resistance load while engaging in an exergame, the rating of perceived exertion is used in the Exergame Grading Scheme. Three items with the OMNI Resistance Exercise Scale operationalize the perceived exertion for the three major muscle groups: the upper extremities (item 4: PE-UE), the lower extremities (item 5: PE-LE), and the trunk (item 6: PE-Trunk) [34]. The OMNI Resistance Exercise Scale elicits subjective feelings of exertion and ranges from 0 to 10, with words anchored at “0” (extremely easy), “2” (easy), “4” (somewhat easy), “6” (somewhat hard), “8” (hard), and “10” (extremely hard). The 0–10 scale is marked on a slope, with “0” at the lowest point and “10” at the highest point. Four pictorial descriptors showing a “weight lifter” are positioned along the response range consonant with corresponding verbal descriptors. The validity and reliability of this scale have been previously documented [34]. Its concurrent validity for the active muscle group has been well demonstrated by correlations with weight lift (*r* = 0.89, *p* < 0.01) and blood lactic acid concentration (*r* = 0.87, *p* < 0.01).

*Muscular Endurance (Item 7).* Muscular endurance is the second component of muscular fitness. Its conceptual definition is the ability of muscle groups to repeatedly perform muscular contractions over a period of time [29]. In the Exergame Grading Scheme, muscular endurance is operationalized as the number of repetitions of muscular contractions instructed by an exergame (item 7: ME). The number of repetitions under the default setting is documented in the Exergame Grading Scheme. This number can be obtained from the exergame software and by observing the number of rounds in an attempt during an exergame. The repetition would increase in an exergame decided by the Wii Fit U software as a user's fitness capability improves.

*Flexibility (Items 8, 9, 10, and 11).* The conceptual definition of flexibility is the range of motion (ROM) at a joint [29]. A joint's functional ability is evaluated by its movement through the full range of motion. In the Exergame Grading Scheme, four major joints in the extremities are considered: shoulders (item 8: ROM-Shoulder), elbows (item 9: ROM-Elbow), hips (item 10: ROM-Hip), and knees (item 11: ROM-Knee). The full distance and directions of ROM for each major joint vary. The greatest distance of ROM in a certain direction for a major joint is determined in the Exergame Grading Scheme. Half of full ROM is decided as the cut-off point for grading purposes. A visual estimate is used to decide whether the active ROM for a joint passes its cut-off point during the exergame. Pictures of full ROM in all possible directions for each major joint can be offered to assist the visual estimation. The grading for flexibility is as follows: “no motion” (Grade 0); “less than half of full active ROM” (Grade 1); and “equal to or greater than half of full active ROM” (Grade 2). In a study, the intraclass correlation coefficients for the knee ROM measurements obtained with a goniometer and by visual estimation were from 0.84 to 0.94, and the interrater reliability for measurements by visual estimation was 0.83 for knee flexion and 0.82 for knee extension [35].

*Balance (Items 12 and 13).* Balance is a skill-related physical fitness attribute. It is conceptualized as the equilibrium associated with two types of forces: (a) destabilizing force, the force needed for the center of mass to reach and stop at the limit of the base of support (postural aspect of balance), and (b) stabilizing force, the force needed to stop the displacements of the center of mass at the limit of the base of support (dynamic aspect of balance) [23]. Exergames with greater changes in the base of support and center of mass can stimulate balance control enough to bring about an improvement in balance. A study found that the level of challenge for balance during two Wii Fit exergames, “Ski Slalom” and “Soccer Heading,” was similar to the gait at natural speed, which means these two exergames challenge the postural aspect of balance (destabilizing force) [23]. In the Exergame Grading Scheme, two items are used to operationalize the concept of balance: “narrowing the base of support” and “displacing the center of mass.” The grading system, based on* ACSM's Resource Manual for Guidelines for Exercise Testing and Prescription *[29], shows the progressive challenges to the body from stressors of increasing difficulty in these two categories. The item for narrowing the base of support (item 12: BOS) includes “sitting” (Grade 0), “feet apart with assistive device” (Grade 1), “feet apart without assistive device” (Grade 2), “feet together” ([tandem; touch along entire length of the feet] Grade 3), “semi tandem stand” ([feet touching but the toe of one foot is at the instep of the other foot] Grade 4), “heel-to-toe stand” ([the toe of one foot is touching the heel of the foot in front] Grade 5), “one-legged stand” ([one foot only is on the ground] Grade 6), “toe stand” ([standing on tip-toe with both or only one foot] Grade 7), and “heel stand” ([standing on both heels] Grade 8). The item for displacing the center of mass (item 13: COM) includes “sitting or standing without moving” (Grade 0), “sitting with the body moving forward, backward, and/or to each side” (Grade 1), “walking” (Grade 2), “standing with feet apart leaning forward and backward, to each side, and/or up and down without moving the feet to maintain the balance” (Grade 3), “turning in a circle” (Grade 4), “shifting body weight from side to side with moving the feet to maintain balance” (Grade 5), “stepping over obstacles, such as a step board” (Grade 6), “turning or leaning while holding a heavy object (such as a book or dumbbell) out in front of the chest” (Grade 7), “crossover walking, sideways walking, heel walking” (Grade 8), “moving weighted arms or legs out to the front or side as in standing resistive exercises with free weights” (Grade 9), and “balancing on a large ball or rocker platform” (Grade 10).

*Cognitive Demand (Item 14).* Cognitive demand is conceptually defined as the thinking process that is required to successfully engage in a task [36]. In the Exergame Grading Scheme, cognitive demand (item 14: CD) is operationalized by either a single motor task (Grade 1) or a cognitive-motor dual task (Grade 2) [12]. A single motor task in the exergame teaches the physical activity “step by step” by a virtual personal trainer. The cognitive-motor dual task is when the exergame requires the attention to be divided between the motor tasks and cognition. A study compared Parkinson's disease patients with healthy older adults; both groups were trained for 10 Wii Fit exergames over 7 weeks [12]. The patients with Parkinson's disease showed no deficit in learning and retention in 7 of 10 exergames although their performance was not comparable to that of the healthy older adults. The three exergames in which they failed to improve (“Obstacle Course,” “Soccer Heading,” and “Basic Run Plus”) required engaging in cognitive-motor dual tasks, including motor activities with stimulus-based decision making, response inhibition to a virtual threat, divided attention to multiple stimuli, and/or working memory. The researchers concluded that dopamine depletion may influence cognitive function in patients with Parkinson's disease, thus affecting cognitive-motor dual task skills.

*Scoring Procedure.* The grade (i.e., scoring mechanism) of the Exergame Grading Scheme was determined using the following procedure. The user operates an exergame while the rater obtains information from the exergame system, observes the exergame physical activity performed by the user, and assesses the user's level of exertion. Using an Exergame Grading Grid with the list of exergames, the rater documents the grading scores from the Exergame Grading Scheme for the corresponding exergame.

#### 2.1.2. Stage II: Quantitative Evaluation of Content Validity

*Participants.* The CVI was based on a quantitative judgment of item relevance by content experts. A minimum of three content experts has been suggested by Lynn [28]. To be content experts in the current study, professionals were required to have a specialty in exercise or rehabilitation science and to have experience publishing or presenting studies in research journals or at conferences.

*Procedure.* The content experts were nominated by the research team and invited to participate in the study. They received a survey link through e-mail. The survey was built in online survey software supported by the University (Qualtrics, Provo, UT, USA). Each content expert responded to and submitted the survey without any personal identification.

On the survey, a total of 14 conceptual definitions related to items on the Exergame Grading Scheme were provided. The content experts were asked to rate the relevance of the grading description (operational definition) for the corresponding conceptual definition. They were instructed to answer “yes, relevant” (score 1), “no, not relevant” (score −1), or “unsure of the relevance” (score 0) [26, 37]. Content experts could provide additional feedback on a specific item using a comment column.

*Statistical Analyses.* The item-level content validity index (I-CVI) was evaluated in this study [26, 37]. I-CVI is computed as the sum of the relevance scores divided by the total number of content experts for an item. I-CVI ranges from 1 to −1. A higher score indicates greater agreement among content experts on an item. To decrease the possibility of an inflated value from a chance agreement, kappa statistics (*k*) [26] were included in the quantitative evaluation of the content validity in the current study. CVI is considered “excellent” when *k* is above 0.74; CVI is “good” when *k* is between 0.60 and 0.74. Items with *k* values of less than 0.74 would need to be revised. In this case, a second round of content expert reviews would be necessary. Analyses were conducted using Microsoft Excel (Microsoft, Redmond, WA, USA).

### 2.2. Interrater Reliability

#### 2.2.1. Participants

The IRR was the strength of the consistency between the two raters who used the Exergame Grading Scheme to determine the grading score on a Wii Fit U exergame performed by two users [38]. The literature suggests having two competent raters is sufficient [39]. Therefore, for this study, two oncology healthcare professionals were trained to use the Exergame Grading Scheme. There were two phases of rater training for using the developed scale. First, the two raters were instructed on how to use the Wii Fit U and the Exergame Grading Scheme. Second, they used the Exergame Grading Scheme to grade a Wii Fit U exergame while a healthy adult engaged in the exergame physical activity. The results of the grading were discussed after each exergame was performed. The goal of the training with the healthy adult was to reach 100% agreement between the two raters on two consecutive exergames.

The two users were cancer survivors who were recruited from a head and neck cancer clinic at a cancer center by the convenience sampling approach. They met the following criteria: They had finished their cancer treatments (completed chemotherapy or radiation or had a month after cancer surgery); were 18 years or older; were able to understand English; had an Eastern Cooperative Oncology Group (ECOG) Performance Status grade of ≤2; and had been cleared by their provider to resume low to moderate intensity physical activities. An ECOG Performance Status grade of 2 describes an individual who is ambulatory and capable of all self-care but is unable to carry out any work activities [40]. The lower the ECOG grade, the more physically fit the individual. Cancer survivors were excluded if they were hospitalized; were in hospice care; were pregnant; had a cardiac pacemaker; had a history of seizure or loss of consciousness; or were cognitively impaired, defined as three or more errors on a validated 6-item cognitive screener [41].

#### 2.2.2. Procedure

During the test, one exergame was selected from each of the five activity categories designated by Wii Fit U: “Warrior” from the category of yoga, “Rowing Squat” from the category of strength training, “Basic Run” from the category of aerobics, “Beginner Dance” from the category of dance, and “Skip Jump” from the category of balance. These exergames were chosen because they were frequently prescribed to cancer survivors in a feasibility study [42]. The two users completed a brief demographic and clinical survey; then they were instructed on how to operate Wii Fi U. The two users performed five exergames randomly in a row while the two raters used the Exergame Grading Scheme to decide the grading score for each exergame based on each of the 14 items.

#### 2.2.3. Statistical Analyses

Descriptive statistics were used to describe the demographic and clinical variables of the two users as well as items from the Exergame Grading Scheme. The purpose for evaluating IRR was to understand if raters could consistently apply the scoring mechanism of the Exergame Grading Scheme [43]. Spearman's rank correlation (rho) was utilized to evaluate the consistency between the two raters' grading scores on the five exergames. Analyses were conducted with SPSS (IBM, Armonk, NY, USA).

## 3. Results

### 3.1. Quantitative Evaluation of Content Validity

There were two rounds of quantitative evaluation of the content validity in order to support the first hypothesis. The results of I-CVI and *k* are shown in Table 1. In the first round, the five content experts provided their scores on item relevance. Five out of 14 items had 1 for I-CVI and for *k*. This means that all five content experts thought these items were relevant to their conceptual definitions. These items, in the domains of “energy expenditure” and “flexibility,” were considered to have excellent content validity. Seven items had 0.8 for I-CVI and 0.76 for *k*. An I-CVI value of 0.8 means that four out of the five content experts agreed on the relevance of the item with its conceptual definition, while one of the experts was unsure about the relevance. Their *k* values were within the excellent range (>0.74). These 7 items were under the domains of “cardiorespiratory fitness,” “muscle strength,” “muscle endurance,” and “cognitive demand.” The one content expert who did not agree on the relevance thought the wording in these items was unclear. Unexpectedly, the 2 items in the “balance” domain only had 0.6 for I-CVI and 0.42 for *k*. Two of the five content experts were unsure about the relevance of these items related to their conceptual definition. The *k* value indicates that their content validity was fair. The suggestions for these two items by the content experts were on revision of the wording but not elimination. In response, we modified the wording to clarify the grading descriptions for the items that did not have full agreement among the five content experts (i.e., I-CVI ≠ 1).

According to Polit et al. [26], a second round of evaluation is needed for any alteration in the instrument after the qualitative evaluation of the content validity. For this reason, we did a second round of evaluation after revising the items that did not receive full agreement. In this round, three different content experts evaluated the item relevance of the Exergame Grading Scheme. Their responses showed that each of the 14 items had a score of 1 for both I-CVI and *k*. This result supports the first hypothesis that all the items in the scheme had excellent content validity. Example items from the finalized Exergame Grading Scheme are shown in Table 2.

### 3.2. Interrater Reliability

Two trained oncology healthcare professionals rated the two users during the performance of five exergames for 10 assessments of each item. Two males, both of whom had oral cancer, participated as users in the study. Participant A was 75 years old with stage II cancer treated with surgery and was 17 months from his last cancer treatment. His body weight was 160 lb and his body height was 5 feet 8 inches. Participant B was 28 years old and had stage I cancer treated with surgery and radiation and was 3 months from his last cancer treatment. He weighed 220 pounds and was 5 feet 11 inches tall. The results of the IRR are shown in Table 3. Four items had rho values between 0.80 and 0.89 (ME, ROM-Knee, COM, and CD). One item had 0.93 (ROM-Elbow). Nine items had 1 (MET, HRR, PE-CR, PE-UE, PE-LE, PE-Truck, ROM-Shoulder, ROM-Hip, and BOS). The second hypothesis in the current study was supported by the rho values for all the items in the Exergame Grading Scheme, which were above 0.80.

## 4. Discussion

The psychometric evaluations of the Exergame Grading Scheme have met the criteria initially hypothesized. For CVI, comments from the first round of the quantitative evaluation of the content validity did not suggest deleting any items; instead, they focused mainly on modification and clarification of the items and their grading descriptions. After the revision, the second round of evaluation had consistent agreement on item relevance with the conceptual definition among the 14 items on the Exergame Grading Scheme. The finalized Exergame Grading Scheme had an acceptable IRR of rho values between 0.82 and 1.

The Exergame Grading Scheme developed in this study offers some significant advantages over similar instruments. Deutsch's instrument was designed to analyze Wii Sport and Wii Fit exergames for stroke rehabilitation [25]. Chen's instrument was designed to analyze the exergames in PlayStation (Sony Interactive Entertainment LLC, San Mateo, CA, USA) for the purpose of upper extremity rehabilitation [44]. Guo's instrument was designed as a content evaluation guideline for fitness professionals who use their knowledge to judge the appropriateness of mobile applications for fitness training purpose [45]. The Exergame Grading Scheme has much better usability than other instruments. First, the Exergame Grading Scheme has been developed based on the physiological attributes of human movement (i.e., energy expenditure and physical fitness attributes) and the cognitive demand described in the gaming literature. Therefore, the Exergame Grading Scheme can easily be used in various populations with chronic conditions and across different types of interfaces, such as Nintendo Switch (Nintendo, Redmond, WA, USA), Xbox (Microsoft, Redmond, WA, USA), PlayStation, or mobile applications. Second, the Exergame Grading Scheme has a comprehensive grading system to operationalize multidimensional body movements during exergame activities, not just categorizing of motor functioning related to stroke rehabilitation as in Deutsch's instrument or classifying of upper extremity mobility as in Chen's instrument. The energy expenditure and physical fitness attributes defined in the Exergame Grading Scheme can be measured objectively and the grading system can be used to rank the training intensity of a physical fitness attribute in an exergame. In contrast, Guo's instrument relies on subjective clinical judgment to determine the appropriateness of the fitness safety and training principles in the content of mobile applications.

While it offers several advantages, the Exergame Grading Scheme should be used with caution for several reasons. First, for the domain of energy expenditure, we currently propose using the number of METs provided by Wii Fit U in the Exergame Grading Scheme. These MET numbers are more likely generated from healthy adults [32]. Based on the literature, METs vary by age and health condition. When playing the same exergame, children had greater METs than adults [46]; adults had greater METs than older people [47]; functionally capable individuals had greater METs than those who had had a stroke [48]; and those with healthy weight had greater METs than those who were obese [49]. Therefore, the MET shown in the exergame should be considered as a relative number but not an absolute one. Second, for the domain of cardiorespiratory fitness, we chose to use HRR% instead of VO_2max_ because of its feasibility and accuracy for the population with chronic conditions [29]. For the same reason, the Adult OMNI Scale of Perceived Exertion for the Walking/Running Exercise selected in the Exergame Grading Scheme contains an easy scoring system from 0 to 10, while the traditional Borg scale goes from 6 to 20 [50]. Third, for the domain of muscular strength, one must be aware that scores from the 0–10 OMNI Resistance Exercise Scale only provide information on the intensity of the exergame physical activity in relation to muscular strength; these scores do not serve as a direct indicator of muscle strength. Fourth, for the domain of muscular endurance, similar to muscular strength, the number of muscular contractions is not a direct indicator of muscle endurance. However, documenting repetitions involved in an exergame activity can give an indication of the challenge of the exergame and can thus provide an idea of how this exergame activity might affect muscle endurance if it is done regularly. In Table 3, the item on ME does not include analyses of the “Basic Run” exergame because this exergame contains rapid lower extremity muscle contraction. We suggest using a pedometer/accelerometer to measure the step count in order to obtain the number of repetitions for the lower extremity activity. Fifth, for the domain of flexibility, the Exergame Grading Scheme uses half of ROM as the cut-off point, which is not a direct measure of flexibility. Instead, the grading system for the domain of flexibility gives an indication of how playing the exergame might improve flexibility. Sixth, for the domains of balance and cognitive demand, the items were developed directly from theoretical concepts. Future revisions may, therefore, be necessary after more advanced psychometric evaluation is done.

The current study has several strengths. We used theoretical constructs in human movement and the exergame literature in developing the Exergame Grading Scheme. The content validity was developed based on Lynn's rigorous method with quantitative evaluation of CVI. The IRR was built on two competent raters and two cancer survivors for five exergames. Both the results of CVI and of IRR supported the proposed hypotheses and were scientifically acceptable. However, more advanced evaluation is still needed to strengthen the psychometric properties. An adequate sample size in the targeted population would allow for factor analysis, which would confirm the seven domains in the Exergame Grading Scheme. Many items in the scheme do not measure actual domain construct but rather provide relative values that indicate potential improvement in the domain if an individual practices an exergame for a certain period of time. Therefore, use of adequate scientific methods to measure the domain constructs is suggested. For energy expenditure, a portable telemetric gas analysis system [48] to measure the METs could be used instead of taking the number from Wii Fit U. ActiGraph wGT3X-BT (Actigraph LLC, Pensacola, FL) is considered to be a user friendly, objective, and reliable/valid measure technology to calculate energy expenditure [51]. For flexibility, use of motion analysis technology is possible to capture the actual active ROM for each joint. Another issue is using the default number of repetitions for muscular endurance, especially in the strength training and yoga exergames of Wii Fit U. Unlocking the default setting to measure muscular endurance for grading purposes in the Exergame Grading Scheme remains to be determined and requires further study. In terms of reliability, a test-retest reliability with a larger number of assessments of the exergames is suggested. In particular, we only included two head and neck cancer patients in the reliability testing, and they might not be representative of the general cancer populations. Tests performed with different age ranges, with both gender groups, and with various functions, symptoms, and disease conditions are warranted. Finally, future revision of the Exergame Grading Scheme needs to take into account possible disabilities or complications from cancer disease and treatment. Examples may include lymphedema after breast cancer and head and neck cancer surgeries or chemotherapy induced cognitive dysfunction.

## 5. Conclusion

The valid and reliable Exergame Grading Scheme makes it possible to develop a personalized physical activity program using exergames in rehabilitation practice. A fitness test measuring a specific physical fitness attribute (i.e., a domain in the Exergame Grading Scheme), such as a hand grip test using a dynamometer for upper extremity muscular strength, provides a parameter for choosing exergames based on the grading scores in the Exergame Grading Scheme. If an exergame selection can meet an individual's fitness parameter, it is possible to improve his/her confidence to complete tasks of physical activity (i.e., task self-efficacy), and then he/she is more likely to continuously engage in physical activity behavior. More importantly, the relationship between task self-efficacy and physical activity behavior is reciprocal [52]. Before-physical activity task self-efficacy has a positive relationship with physical activity behavior. From the experience of mastery and performance accomplishment, physical activity behavior is positively related to after-physical activity task self-efficacy as well. Constant high task self-efficacy leads to continuous physical activity behavior. Task self-efficacy has been demonstrated to be an important determinant for initiating and maintaining physical activity behavior in cancer survivors [53]. Therefore, the Exergame Grading Scheme offers an innovative rehabilitation strategy that has great potential to improve physical activity behavior among cancer survivors.

## Figures and Tables

**Table 1:** Content validity index of Exergame Grading Scheme.

Domain: item	CVI first round (*N*^a^ = 5)			CVI second round (*N*^a^ = 3)		
	I-CVI	*k*	Evaluation^b^	I-CVI	*k*	Evaluation^b^
Energy expenditure: MET	1.00	1	Excellent	1	1	Excellent
Cardiorespiratory fitness: HRR%	0.80	0.76	Excellent	1	1	Excellent
Cardiorespiratory fitness: PE-CR	0.80	0.76	Excellent	1	1	Excellent
Muscular strength: PE-UE	0.80	0.76	Excellent	1	1	Excellent
Muscular strength: PE-LE	0.80	0.76	Excellent	1	1	Excellent
Muscular strength: PE-Trunk	0.80	0.76	Excellent	1	1	Excellent
Muscular endurance: ME	0.80	0.76	Excellent	1	1	Excellent
Flexibility: ROM-Shoulder	1.00	1.00	Excellent	1	1	Excellent
Flexibility: ROM-Elbow	1.00	1.00	Excellent	1	1	Excellent
Flexibility: ROM-Hip	1.00	1.00	Excellent	1	1	Excellent
Flexibility: ROM-Knee	1.00	1.00	Excellent	1	1	Excellent
Balance: BOS	0.60	0.42	Fair	1	1	Excellent
Balance: COM	0.60	0.42	Fair	1	1	Excellent
Cognitive demand: CD	0.80	0.76	Excellent	1	1	Excellent

BSO: narrowing the base of support; CD: cognitive demand; COM: displacing the center of mass; CR: cardiorespiratory fitness; CVI: content validity index; HRR%: percentage of heart rate reserve; I-CVI: item-level content validity index; IRR: interrater reliability; *k*: kappa statistics; LE: lower extremities; ME: muscular endurance; MET: Metabolic Equivalent of Task; PE: perceived exertion; ROM: range of motion; UE: upper extremities. ^a^Number of content experts. ^b^Evaluation criteria: fair = *k* of 0.40 to 0.59; good = *k* of 0.60 to 0.74; excellent = *k* more than 0.74.

**Table 2:** Example of items in Exergame Grading Scheme (Federal Copyright Registration: TXu 1-996-913).

Items Grading methods	Grading numbers	Grading descriptions
(1) Energy expenditure Document numbers of METs shown on an exergame.	#	Numbers of METs

(2) Cardiorespiratory fitness: percentage of heart rate reserve (HRR) Use the user's age, his/her resting heart rate, and peak heart rate during an exergame to calculate the HRR.	0–100	Use the following formula to calculate the percentage of HRR: (peak heart rate − resting heart rate) ÷ (220 – age – resting heart rate) × 100%

(3) Cardiorespiratory fitness: perceived exertion Use the Adult OMNI Scale of Perceived Exertion for the Walking/Running Exercise to ask about feelings of exertion during an exergame.	0–10	The user reports a perceived peak maximal exertion score that is consonant with the score depicted visually by the figure walking/running at the bottom (rating 0) and top (rating 10) of the hill, as presented in the Adult OMNI Scale of Perceived Exertion for the Walking/Running Exercise

**Table 3:** Interrater reliability of Exergame Grading Scheme (*N*^a^ = 10).

Domain: item	Rater 1			Rater 2			IRR
	*M*	SD	Range	*M*	SD	Range	rho
Energy expenditure: MET^b^	3	1.20	2–5	3	1.20	2–5	1
Cardiorespiratory fitness: HRR%	121.50	14.28	102–148	121.50	14.28	102–148	1
Cardiorespiratory fitness: PE-CR	3.40	1.50	1–5	3.40	1.50	1–5	1
Muscular strength: PE-UE	1.90	0.74	1–3	1.90	0.74	1–3	1
Muscular strength: PE-LE	3.70	1.70	1–6	3.70	1.70	1–6	1
Muscular strength: PE-Trunk	1.90	0.74	1–3	1.90	0.74	1–3	1
Muscular endurance: ME^c^	6.63	5.07	1–15	7.88	5.79	1–15	0.86
Flexibility: ROM-Shoulder	1.10	0.74	0–2	1.10	0.74	0–2	1
Flexibility: ROM-Elbow	1.00	0.82	0–2	1.10	0.74	0–2	0.93
Flexibility: ROM-Hip	1.10	0.32	1-2	1.10	0.32	1-2	1
Flexibility: ROM-Knee	1.20	0.42	1-2	1.10	0.57	0–2	0.86
Balance: BOS	2.4	1.26	2–6	2.40	1.26	2–6	1
Balance: COM	3.20	1.03	2–5	3.5	1.35	2–6	0.83
Cognitive demand: CD	1.6	0.52	1-2	1.5	0.53	1-2	0.82

BSO: narrowing the base of support; CD: cognitive demand; COM: displacing the center of mass; CR: cardiorespiratory fitness; HRR%: percentage of heart rate reserve; LE: lower extremities; *M*: mean; ME: muscular endurance; MET: Metabolic Equivalent of Task; PE: perceived exertion; ROM: range of motion; SD: standard deviation; rho: Spearman's rank correlation; UE: upper extremities. ^a^Number of rater assessments (2 raters × 5 exergames). ^b^Five exergames were assessed. Their METs were provided by Wii Fit U: “Warrior” (2 METs), “Rowing Squat” (3.5 METs), “Basic Run” (5.0 METs), “Beginner Dance” (2.5 METs), and “Ski Jump” (2.0 METs). ^c^The exergame of “Basic Run” was not included in the calculation.
